# Parsonage–Turner Syndrome following COVID-19 Vaccination: A Systematic Review

**DOI:** 10.3390/vaccines12030306

**Published:** 2024-03-14

**Authors:** Elena Cecilia Rosca, Almonzer Al-Qiami, Amalia Cornea, Mihaela Simu

**Affiliations:** 1 Department of Neurology, Victor Babes University of Medicine and Pharmacy Timisoara, 300041 Timisoara, Romania; amalia.cornea@umft.ro (A.C.); simu.mihaela@umft.ro (M.S.); 2 Department of Neurology, Clinical Emergency County Hospital Timisoara, 300736 Timisoara, Romania; 3 Faculty of Medicine and Health Science, University of Kassala, Kassala 1115, Sudan; almonzerm.ahmed@gmail.com

**Keywords:** Parsonage–Turner syndrome, neuralgic amyotrophy, COVID-19, vaccines, systematic review

## Abstract

Background: Parsonage–Turner syndrome (PTS) is an inflammatory condition of the brachial plexus, with more than half of patients presenting a trigger, such as infection or vaccination. Our objective was to synthesize the clinical and paraclinical features, therapeutic responses, and outcomes of PTS post-COVID-19 vaccination. Methods: We systematically reviewed two databases (LitCOVID and the WHO database on COVID-19) up to January 2024 following a published protocol (OSF registries). Results: We included 59 cases. PTS occurred more frequently in males (61.1% mRNA group, 83.3% viral vector group). Patients in the mRNA group were younger (41.7% between 41 and 50 years vs. 38.9% between 61 and 70 years). Most cases had sudden pain within two weeks. Unilateral PTS was present in 94.4% of mRNA and all viral vector-vaccinated cases. Symptoms included pain (97.1% and 92.3%, respectively), usually followed within two weeks by motor deficits (97.2% and 94.1%, respectively), amyotrophy (30% and 81.8%, respectively), paresthesia (50% and 27.3%, respectively), and sensory loss (33.3% and 38.5%, respectively). Viral vector vaccine recipients had nerve involvement outside the brachial plexus. Ancillary investigations revealed CSF albuminocytological dissociation (33.3% and 100%, respectively) and ipsilateral axillary lymphadenopathy. Two PTS cases worsened after the second mRNA dose, and another recurred after influenza vaccination. One patient well tolerated the second dose of the viral vector vaccine, but symptoms reemerged in another. Conclusions: Current evidence suggests PTS may occur after all COVID-19 vaccine types, with some subgroup differences. Also, PTS might recur with subsequent similar or unrelated vaccines.

## 1. Introduction

Parsonage–Turner syndrome (PTS), also known as neuralgic amyotrophy, is a disorder involving the peripheral nervous system characterized by intense pain and significant muscular atrophy. The symptoms mainly impact the forequarters of the body, such as the cranium, shoulder, upper limb regions, and ipsilateral side of the chest wall [1].

During the mid-1800s, clinicians identified two distinct conditions, serratus magnus paralysis and post-infectious paralysis, characterized by the involvement of the serratus anterior muscle and their occurrence after an infection. Later, two other conditions were documented, serogenic neuropathy and vaccinogenic neuropathy, caused by specific triggers. Subsequently, further entities were recognized and categorized using terms relevant to their location, pathology, or cause. In 1948, Parsonage and Turner identified the shared attributes of these diseases, ultimately specifying a unified entity with diverse manifestations [2]. The term “neuralgic amyotrophy” was established by recognizing two prominent clinical characteristics: intense pain and notable muscular atrophy. A unifying clinical triad was determined: a preceding incident or stimulus, abrupt onset of acute pain in the upper body area, and significant weakness and atrophy of nearby muscles, with widespread recognition that these conditions are different expressions of the same disease.

Neuralgic amyotrophy is a relatively uncommon condition, with an estimated annual incidence of 1.64 cases per 100,000 people [3]. However, the incidence is expected to be significantly higher because it is under-recognized. One prospective study estimated an incidence rate of one case per 1000 population, approximately 60-fold higher than the previous epidemiological data [4].

At least 50% of PTS attacks are associated with triggering events, the most common being an upper respiratory tract infection. Nonetheless, a prospective approach identified a trigger in 73% of patients, including medical or surgical procedures (29%), upper respiratory infections or nonspecific flulike infections (24%), excessive or unaccustomed physical activity (17%), closed trauma (10%), delivery (7%), dental procedures (6%), immunization (5%), and open traumatisms (2%) [5]. 

Several bacterial and parasitic infections were reported to trigger PTS, including pneumonia, rheumatic fever, diphtheria, dysentery, typhus, malaria, borreliosis, and sepsis. Also, viruses (i.e., influenza, cytomegalovirus, hepatitis B, herpes virus, varicella-zoster virus, smallpox, Epstein–Barr, parvovirus B19, coxsackie, Echo 13/30 virus, and poliomyelitis) have been identified as potential triggers of PTS [6]. Furthermore, about 10% of patients with PTS presented simultaneous infection with the hepatitis E virus in the acute phase, explaining prior observations of increased levels of liver enzymes in some cases [7]. Additional factors include immunization and vaccination, surgical or medical procedures, childbirth, sudden physical exertion, and trauma. The trauma can be as minor as a fall without any visible injury or as a result of intravenous procedures (e.g., blood withdrawal, intravenous therapy, contrast injections) [5]. Furthermore, PTS has been documented after the administration of some drugs, such as nivolumab [8] and botulinum toxin [9].

The latency period between the trigger event and the onset of PTS is typically defined as 4 to 6 weeks in duration. In a large cohort of PTS, the latency period varied from a few hours to 28 days, and in 67% of cases, pain started during the first week [5].

The majority of triggering circumstances suggest an underlying autoimmune disease characterized by specific inflammation of the peripheral nerves. In addition, nerve biopsies in cases of acute PTS reveal the existence of lymphocytic inflammatory infiltrates in the afflicted nerves. The initial inflammation results in intraneural edema, reducing the flexibility of the fascicles. The movement of a neighboring joint causes bending or folding, with repeated kinking and twisting of the nerves that cause narrowing and fascicular entwinement. Several patients have reported engaging in vigorous physical activity involving the upper body before PTS onset. Therefore, mechanical stress may have a predisposing role [7]. Repetitive microtrauma to the nerves may cause increased blood–nerve barrier permeability, allowing immune factors to enter the endoneurial region and facilitating the autoimmune process [7].

The abrupt onset of PTS, the monophasic course, and the association with prior infections, serum sickness, vaccination, or immunomodulating medications all provide evidence for immune-mediated pathology. This hypothesis is reinforced by the participation of both humoral and cellular immune processes, as well as the existence of focal chronic inflammatory infiltrates, edema, and the characteristic onion bulb appearance. Mononuclear inflammatory infiltrates surround the endoneurial and epineurial vessels, but there are no signs of necrotizing vasculitis [5]. 

Patients with PTS were found to have altered lymphocyte subsets (specifically, lower levels of CD3 and elevated CD4/CD8 ratios as a result of decreasing CD8 levels), antiganglioside and anti-peripheral nerve myelin antibodies, and terminal complement activation products [10,11,12,13]. Oligoclonal bands were detected in the cerebrospinal fluid (CSF) of some patients [10,11,13]. In addition, factors that may trigger PTS, such as infections, may also act as triggers for other autoimmune disorders, including acute and chronic inflammatory demyelinating polyradiculoneuropathy [6].

Recently, PTS was reported after COVID-19 vaccination. To date, 13.500.135.157 vaccine doses have been administered [14]. The administered vaccine types are Messenger RNA (mRNA) vaccines and viral vector vaccines. While worldwide immunization against COVID-19 infection is beneficial, there are still concerns about possible adverse effects. Previous research reported PTS occurrence after SARS-CoV-2 infection. Moreover, a causal association between PTS and COVID-19 vaccination has been suggested. Our objective was to systematically review PTS cases reported after COVID-19 immunization. We aimed to provide an extensive perspective of this pathology and identify further research questions that could be addressed more specifically. Furthermore, our objective was to emphasize research gaps that require further investigations. Therefore, our goals were to evaluate the clinical, laboratory, neurophysiological, and neuroimaging features of PTS following COVID-19 vaccination, to explore its potential association, and to understand how it differs from the typical PTS manifestations with the ultimate purpose of prompt identification and appropriate management of at-risk individuals. 

## 2. Materials and Methods

We followed a published protocol (OSF Registries https://doi.org/10.17605/OSF.IO/PKFV8, accessed on 2 March 2024) in accordance with the guidelines of the Preferred Reporting Items for Systematic Reviews and Meta-Analyses extension for Scoping Reviews (PRISMA-ScR) [15,16,17] and the current recommendations on the synthesis of case reports and case series [18]. We defined our research questions based on the Population, Concept, and Context (PCC) of the review [15]:

Is there a relationship between COVID-19 vaccination and the development of PTS?
If yes, what are the clinical features?
What do we know about additional investigations?
What are the presumptive mechanisms underlying PTS?
Which interventions might be effective?
What do we know about the evolution of PTS after COVID-19 vaccination?

We searched LitCOVID and the World Health Organization database on COVID-19 (to 25 January 2024) using the following search terms: “Parsonage AND Turner” and “brachial”. As these databases are curated for COVID-19 articles, we did not need to use search terms like “coronavirus,” “COVID-19”, or “SARS-CoV-2”. Additional studies were searched using the reference lists of relevant articles. As we aimed to generate an extensive list of publications suitable for answering our questions, no search filters or language restrictions were applied. The screening and selection of papers were conducted by one reviewer and cross-checked by a second author. Disagreements were managed by discussions between the two screeners. Two authors reviewed the full text of all retrieved studies, assessing whether they met the inclusion criteria. A third reviewer’s opinion was considered if disagreements were not solved through discussion.

The PCC mnemonics for this systematic review were patients of all ages (children and adults) (P), with studies investigating patients with PTS (C) in the context of previous COVID-19 vaccination (C). We included case reports and case series, as well as prospective or retrospective observational and interventional studies. We also included conference abstracts when the authors did not publish a full article on the topic. Commentaries, opinions, and narrative reviews were excluded, but we carefully evaluated their reference lists to identify potential additions.

The data were retrieved using a pro forma template piloted on a sample of five randomly selected articles. The template was then updated as necessary. The data were retrieved by one reviewer, while a second reviewer verified the data. We did not formally evaluate the methodological quality of the included studies, as our primary scope was to provide an overview of the evidence reported on PTS triggered by COVID-19 vaccination, regardless of the risk of bias in the included studies [15]. However, post hoc, we decided to use the WHO-UMC causality assessment system [19] to investigate the connection between the administration of the SARS-CoV-2 vaccine and PTS. Each case was evaluated independently by two authors utilizing the WHO-UMC system. If there were any disagreements, a third author arbitrated. 

Also, our causality evaluation was guided by the basic framework suggested by the WHO to develop an adverse event following immunization (AEFI)-specific causality assessment [20]. In our assessment, as the Brighton Collaboration criteria [21] do not define PTS, we used the symptoms and exclusion criteria proposed to diagnose PTS [4,6]. For alternative diagnoses, we used previously published lists of the precipitating conditions [4,6]. 

We performed descriptive statistics, presenting patient demographics, clinical characteristics, ancillary investigations, treatments, and outcomes. Categorical variables are presented as numbers (percentages). In further analysis, differences for subgroups of mRNA vaccines and viral vector vaccines were tested for categorical variables using Fisher’s exact test (two-tailed testing for a significance level of *p* < 0.05).

## 3. Results

**Figure 1:**
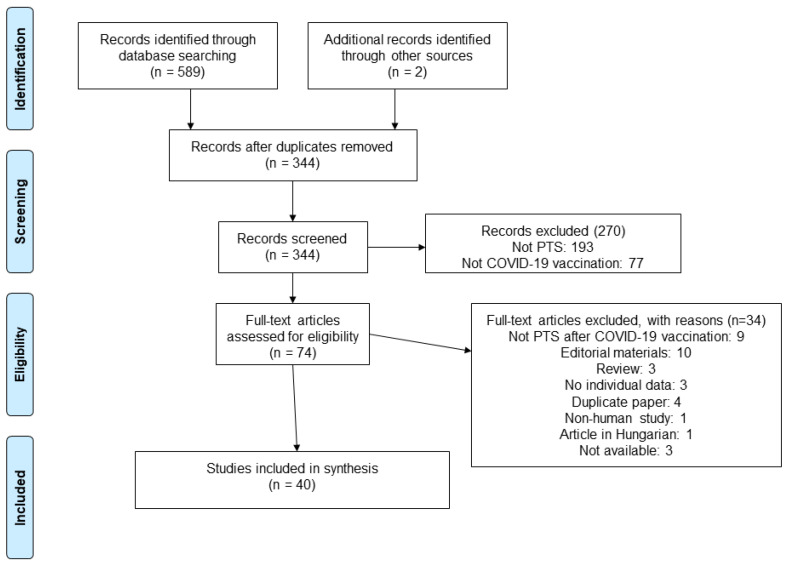
Flow chart showing the process for inclusion of studies.

The literature search resulted in 589 records. After deduplication, 344 articles were included for the title- and abstract-screening phases of the systematic review. Finally, we identified 74 papers on PTS in patients with previous COVID-19 vaccination to assess in full text, and 40 articles were ultimately included; the reasons for exclusion are noted in the PRISMA chart (Figure 1). 

This systematic review included 30 articles [22,23,24,25,26,27,28,29,30,31,32,33,34,35,36,37,38,39,40,41,42,43,44,45,46,47,48,49,50,51] and 10 meeting abstracts [52,53,54,55,56,57,58,59,60,61] reporting on 59 cases. Among them, 36 (61.0%) patients received a mRNA vaccine (24 with BNT162b2, Pfizer, 10 with mRNA-1273, Moderna) [22,24,25,26,27,29,30,34,36,37,38,39,40,41,45,46,47,48,50,51,53,56,60] and 18 (30.5%) received a viral vector vaccine (15 with AstraZeneca, 2 with Janssen) [23,28,31,32,33,35,39,42,43,44,49,54,59]; the type of vaccine used was not reported in 5 (10.6%) individuals [52,55,57,58,61]. 

Patients were aged between 14 and 84 years, mostly males (38/59, 64.4%). The majority of patients vaccinated with a mRNA vaccine were aged between 41 and 50 years (40%), while most individuals with viral vector vaccination were aged between 61 and 70 years (41.2%). The demographic characteristics of the patients are presented in Table 1. The detailed study characteristics are presented in Supplemental Material Table S1. 

### 3.1. Medical History and Comorbidities

Among patients receiving mRNA vaccines, 15 (45.5%) developed PTS after the first dose, including a patient with cross-vaccination with Pfizer following the initial AstraZeneca vaccination. Sixteen cases (48.5%) had PTS after the second dose. One patient (3%) developed neurologic symptoms after mRNA booster vaccination; he had completed two previous doses of an inactivated COVID-19 vaccine [51]. Another case (3%) presented PTS after the fourth dose of the vaccine, completed with Moderna; the first three doses were Pfizer vaccines [48]. The dose was not specified in three (8.3%) cases. In the viral vector vaccination group, six (66.7%) presented PTS after the first dose and three (33.3%) after a second dose. Nonetheless, in nine (50%) patients, the authors did not specify if they had previous COVID-19 vaccination. The comorbidities of each group are presented in Table 2. 

### 3.2. Clinical Characteristics 

As presented in Table 3, most patients presented with PTS symptoms within two weeks after vaccination. 

However, the neurologic symptoms developed 15 to 30 days after vaccination in eight (22.2%) cases receiving mRNA vaccines and two (11.8%) receiving a viral vector vaccine. The onset was acute in 25 (86.2%) patients in the mRNA group and 8 (80%) in the viral vector vaccination group. Among patients with previous mRNA vaccination, 34 (94.4%) had unilateral PTS. The neurologic symptoms were ipsilateral to the vaccination site in 21 (75%). On the other hand, all patients with viral vector vaccination had unilateral PTS, mostly with ipsilateral symptoms (11 cases, 91.7%). In addition, in the latter group, one case (5.6%) had lower limb involvement, and two (11.1%) had diaphragmatic paralysis. 

The clinical picture of individual cases is presented in Supplemental Material Table S2. The extended data can be found in Supplemental Materials Table S1. The detailed pain characteristics are presented in Supplemental Material Table S1.

### 3.3. Ancillary Investigations

The data on ancillary investigation results are presented in Table 4 and Supplementary Materials Table S1. 

Lumbar puncture was performed in three patients from the mRNA group, with one case (50%) presenting albuminocytological dissociation [38,39,46]. In the viral vector vaccine group, cerebrospinal fluid (CSF) was analyzed in three cases [39,54], all with albuminocytological dissociation, including a patient with concomitant Guillain Barre syndrome [54]. 

Nerve conduction studies (NCSs) were conducted in 23 (63.9%) patients in the mRNA group and 10 (55.6%) receiving viral vector vaccines. The findings varied depending on the nerve fibers affected and the timing of the investigation. In individuals receiving mRNA, the authors reported subacute plexopathy [24]; neuropathic changes and active denervation [29]; absence of sensory nerve action potential (SNAP) and compound motor action potential (CMAP) [26]; or normal findings in five (21.7%) cases. Also, in PTS following a viral vector vaccine, the findings ranged from normal in one patient (10%) to acute denervation and changes suggesting neurological recovery. Some authors only noted that they performed electrophysiological studies for their patients, all with pathological findings in both subgroups. 

Likewise, the electromyography (EMG), including needle EMG, results were also heterogeneous. In patients receiving mRNA vaccines, authors reported decreased motor unit recruitment [22,37,40,41], abnormal spontaneous activity [56], acute-to-subacute brachial plexopathy [25], fibrillations and positive sharp waves [26,50], chronic neuropathic changes [27], and normal findings [34]. In the other group, the EMG revealed acute denervation signs [23,42,43], fibrillations and positive waves [28,31], decreased motor unit recruitment [39], and reinnervation signs [23,44]. 

Ultrasonography detected ipsilateral axillary lymphadenopathy in one patient receiving mRNA vaccines [39]. Also, chest computed tomography (CT) was employed in documenting diaphragmatic dysfunction [28,32]. 

MRI of the brachial or lumbar plexus was reported for 18 (50%) patients receiving mRNA and 9 (50%) receiving a viral vector vaccine. In the mRNA group, the most common finding was muscle edema (five cases, 27.8%), followed by nerve edema (four cases, 22.2%), hyperintensity of the affected nerves (four cases, 22.2%), local lymphadenopathy (three cases, 22.2%), and hourglass constrictions (two cases, 11.1%). On the other hand, in patients receiving viral vector vaccines, the most frequent findings were local lymphadenopathy (four cases, 44.4%) and muscle edema (four cases, 44.4%), followed by an increased signal of the nerve roots (three cases, 33.3%). 

### 3.4. Interventions and Outcomes

As shown in Table 5, most patients received medical treatment: 91.7% of individuals with PTS following mRNA vaccination and 73.3% of cases with viral vector vaccination.

In both groups, the patients were prescribed various regimens, including corticosteroids [22,24,25,26,28,29,30,33,34,36,37,38,39,41,48,50,53,56,60], nonsteroidal anti-inflammatory drugs (NSAIDs) [25,33,39,40,41,42,43,44,45,49], Pregabalin [26,39,44,48], Gabapentin [24,41,50], and acetaminophen [44,53]. In addition, in the mRNA group, one patient (2.9%) received intravenous immunoglobulin [46], two were prescribed tricyclic antidepressants (5.7%) [39,48], and four (11.4%) received opiates [39,48,53,60]. One patient with minimal recovery at ten months underwent neurolysis of C5 and C6 and the upper trunk (at 46 weeks from symptom onset). Postoperatively, he received ten sessions of low-level laser therapy along the brachial plexus and his status improved [51]. One patient (9.1%) with diaphragmatic dysfunction benefited from continuous positive airway pressure (CPAP) [28]. Physical or occupational therapy was prescribed for patients in both groups (14 cases, 20%; 7 cases, 43.8%, respectively). 

Duration of follow-up varied (Table 5, Supplemental Material Table S1); it was reported for 28 (77.8%) cases receiving a mRNA vaccine and 13 (72.2%) cases in the viral vector group. The outcome of PTS was unclear in three (8.3%) individuals in the mRNA group and six (33.3%) in the viral vector vaccines group. In patients receiving mRNA vaccines, one had a complete remission of symptoms by week 3 [39], two cases had a full recovery after 2 months [47,48], and one was reported to regain full range of motion and strength as well as axillary nerve sensitivity at 1 year [45]. The clinical examination was found to be improved in twenty-eight (84.8%) patients, and one (3%) individual had no residual pain but increased weakness at the 3-month follow-up [41]. In patients with PTS following viral vector COVID-19 vaccines, three (25%) had complete clinical recovery by one week [39], within 2 months [39], and at 3 months consecutively [43]. Improvement of clinical symptoms and signs was reported in nine (75%) cases. Nonetheless, the timing of the follow-up visits was heterogeneous in both groups.

### 3.5. Causality Assessment

On the causality assessment [19], we considered that 32 cases were possibly caused by vaccination (22 cases after mRNA vaccines, 7 cases after viral vector vaccines, and 3 cases with unspecified vaccines). Among these patients, five had a thorough assessment of possible PTS triggers (three cases after mRNA vaccines and two cases after viral vector vaccines) [22,26,32,33,37]. However, they did not meet the WHO-UMC scale criteria on withdrawal (“Response to withdrawal clinically reasonable”) to be marked as “Probable/likely”. Additionally, 26 cases were deemed unassessable or unclassifiable due to insufficient information provided by the authors (14 cases after mRNA vaccines, 10 cases after viral vector vaccines, and 2 cases with unspecified vaccines). The details are presented in the Supplemental Table S3.

## 4. Discussion

After the introduction of COVID-19 immunization programs, researchers have noted the development of PTS following mRNA and viral vector vaccines. In this systematic review, we synthesize the evidence that PTS may occur in patients receiving COVID-19 vaccines, highlighting this temporal association and comparing mRNA and viral vector vaccines. We present in detail the clinical features, ancillary testing results, and outcomes of PTS in this context. 

PTS was reported after both mRNA and viral vector vaccination. It occurred more frequently in males, but the proportions were different. In the mRNA group, 64% of cases were males, similar to other cohorts from the literature, including PTS of all causes [1]. The proportion of males in the viral vector group was higher (83.3%), comparable to the gender distribution reported by a previous systematic review on PTS following SARS-CoV-2 infection [62]. A family history was absent in all patients, but most authors did not report on it.

The patients presenting with PTS following mRNA vaccination were younger, 41.7% of them being between 41 and 50 years, similar to other studies on idiopathic neuralgic amyotrophy [1], while most patients in the viral vector vaccination group were aged between 61 and 70 years (38.9%). The age distribution was among the clinical variables that differed significantly between groups (*p* = 0.0098 for the age group 41–50; 0.0041 for the age group 61–70). More than half of the patients (52.9% and 55.6%, respectively) had no other comorbidities.

Most patients in both subgroups had a sudden onset of pain within two weeks after vaccination, and only one patient was reported to develop neuralgic amyotrophy within a timeframe larger than one month. However, it is less likely that patients with PTS developing after 30 days from vaccination would be suspected to have brachial neuritis due to vaccination. Nonetheless, a latency period occurs between the trigger and symptom onset. In the literature, this time typically lasts from 4 to 6 weeks. Previous studies reported a latency duration from a few hours to 28 days, with pain onset occurring during the first week in 67% of cases [5].

After viral vector vaccination, the symptoms were always unilateral, with 91.7% of cases presenting clinical signs ipsilateral to the vaccination side. In patients receiving mRNA vaccines, the symptoms were bilateral in 5.6% of cases, and 25% of individuals developed PTS contralateral to the vaccination side. These findings differ from the literature on PTS, where 28.5% of cases presented bilateral symptoms [1], but reports on PTS after COVID-19 infection found lower rates, with 12% of cases developing bilateral symptoms [62].

In most cases, further PTS symptoms appeared within two weeks after the onset of pain; only two patients with a history of viral vector vaccination presented motor and sensory signs after three weeks [42] and one month, respectively [44]. These findings contrast the data from the literature, including patients with or without COVID-19, where authors found that in 27.2% [1] and 25% [62] of cases, paresis did not manifest itself until over two weeks later [1].

The most frequent complaint in both subgroups was a motor deficit (97.2% and 94.1%, respectively), followed by severe pain (97.1% and 92.3%, respectively). However, paresthesia was reported more frequently after mRNA vaccination (50% of cases) than after viral vector vaccines (27.3%), but this difference was not statistically significant. Muscle wasting was more frequent in the latter group (81.8% vs. 30%, statistically significant, *p* = 0.0046). In cohorts of patients with PTS, 96.3% experienced typical neuropathic pain, 78.4% had sensory involvement, and muscle atrophy was present in 88.5% of males and 75.4% of females [1]. After the COVID-19 vaccination, sensory symptoms were less frequent, but muscle wasting occurred at similar rates only in the viral vector vaccines group. Sensory loss presented similar rates compared to PTS after SARS-CoV-2 infection [62]. 

Although in the literature, 15.4% of PTS patients presented autonomic nervous system involvement (e.g., vegetative and trophic skin changes, edema, temperature dysregulation) [1], autonomic dysfunction was not reported after COVID-19 vaccination or infection [62]. The development of nerve involvement outside the brachial plexus was documented solely following viral vector immunization, including the lumbosacral plexus [33] and phrenic nerve [28,32]. Lumbosacral PTS was also diagnosed in patients with COVID-19 [62] and hereditary PTS [6]. However, lower limb involvement was not found in patients in large cohorts of sporadic neuralgic amyotrophy [63]. Some authors question if PTS and lower extremity muscle involvement represent the same disorder if the latter is not accompanied by forequarter region weakness [6]. For the case of lumbosacral PTS included in the present review, the authors did not provide data on family history of neurological diseases [33]. 

Diaphragmatic paralysis was reported in 7% of cases of idiopathic neuralgic amyotrophy and up to 14% of hereditary PTS [64]. Also, cases with PTS following COVID-19 were documented [62]. In PTS, phrenic nerve involvement can be unilateral or bilateral, with isolated cases often going unnoticed due to nonspecific symptoms, mild and short-lived complaints, or lack of clinical signs. However, these cases are more likely to be recognized when associated with an antecedent trigger or severe shoulder pain [5,6]. In a study of phrenic neuropathies due to neuralgic amyotrophy, 10 of 17 cases were isolated; only 5/10 reported preceding pain, but all identified an antecedent event [65]. Without a detailed history, five patients might not have been recognized. Proper management is essential when confronted with a unilateral phrenic neuropathy of unknown etiology. In such cases, the diagnosis of PTS must be considered so that appropriate care can be provided.

No cranial nerve palsy was noted in any patient with PTS following vaccination. Although rare, being reported in up to 10% of cases [66], cranial nerve involvement is more frequent in individuals with hereditary PTS [1]. 

CSF analysis was seldom reported. Nonetheless, an albuminocytological dissociation was present in all patients with previous viral vector vaccination. Among three patients receiving mRNA vaccines, two had normal findings [38,46], but the other presented an increased albumin level. Local lymphadenopathy was detected in 16.7% of cases with mRNA vaccination and 44.4% of patients with viral vector vaccination. Four of these patients also had CSF testing: three had albuminocytological dissociation (one with mRNA vaccination and two with viral vector vaccination) [39]; however, one patient with PTS following mRNA had swollen axillary and subclavian lymph nodes but a normal lumbar puncture [46]. Reactive lymphadenopathy is frequently found after COVID-19 vaccines [67,68]. Clinical and subclinical lymphadenopathy is detected mainly by 18F-FDG PET-CT; it was reported in up to 36% of vaccinated individuals up to 10 weeks after immunization, with women and patients over 65 most frequently affected [69]. Unilateral lymphadenopathy has rarely been documented with immunizations like influenza, bacillus Calmette–Guérin, and human papillomavirus vaccines [70,71,72]. However, additional research is required to evaluate if these local immune responses contribute to the development of PTS or whether they are coincidentally detected [39]. 

Although no diagnostic markers exist for PTS, routine blood work is necessary to exclude other neuralgic amyotrophy causes. Nonetheless, in our review, laboratory investigations were reported only for a few cases. 

The diagnosis of PTS was primarily clinical, based on the typical history and neurologic examination. Additional investigations included electrodiagnostic studies, MRI, and ultrasound. Most frequently, the authors used EMG, performed in 72.2% of patients receiving mRNA vaccines and 61.1% of individuals with viral vector vaccination, followed by NCS (63.9% and 55.6%, respectively), MRI of the nerve plexus (50% in both subgroups), and MRI of the spine (55.6% and 33.3%, respectively). 

Electrodiagnostic investigations can detect specific lesions in the peripheral nervous system, such as mononeuropathies and multiple mononeuropathies that primarily affect motor nerves, causing significant damage to one muscle while sparing or minimally affecting others. Although electrodiagnostic studies are the first method to be used in patients suspected of PTS, normal results do not exclude, with certainty, the diagnosis. MRI and ultrasound studies might provide information on individual lesions, bringing additional confirmation when required [73,74,75,76]. While MRI is more effective than ultrasound for imaging the brachial plexus, ultrasonography is particularly useful for extraplexal imaging because it can accurately track the nerves and fascicle courses [5,6]. Since the majority of lesions in PTS occur outside the nerve plexus, ultrasonography has an advantage over MRI. Additional benefits of ultrasonography include enhanced spatial resolution, reduced costs, simplicity of conducting side-to-side comparisons, and the ability to perform real-time examinations [5]. Several authors favor the use of ultrasound owing to the fact that the majority of PTS lesions are extraplexal [63,75]. Additionally, MRI’s limited field of view at a certain resolution hinders the thorough assessment of the peripheral nervous system, potentially leading to false-negative findings [6].

MRI studies have described several focal features in PTS [77], including hourglass-like constrictions, pre- and post-lesion dilations, and bullseye changes. Furthermore, the imaging abnormalities were categorized into four distinct types: incomplete focal, full focal (hourglass), multifocal (string of pearls), and segmental [78]. A recent analysis revealed a significant association between hourglass-like constrictions (on MRI and ultrasound), denervation edema (on MR neurography), and fibrillation potentials (on EMG) in the acute phase of PTS [79]. Also, MR neurography studies revealed that most patients had unilateral involvement; the roots were the most common site of involvement, followed by the trunks, cords, and terminal branches [80]. PTS is often referred to as a brachial plexopathy or brachial plexitis. However, a recent study found that 24 out of 27 MRI exams revealed no abnormalities of the plexus proper, supporting PTS being characterized by single or multiple mononeuropathies [75]. The term “brachial plexitis” may introduce ambiguity and may not focus imaging efforts solely on the plexus proper. Furthermore, it may also preclude more focused imaging of branch nerves outside the plexus or distal peripheral nerves. Researchers suggest avoiding using the term “brachial plexitis” to characterize PTS since precise identification of the lesion location may substantially impact the diagnosis and management of the condition.

Patients received various drug regimens. In total, 91.4% in the mRNA group and 75% in the viral vector group received any drug for their symptoms. Interestingly, no medication was prescribed in 14.3% of patients with mRNA vaccination and 25% of cases with viral vector immunization. Among patients receiving mRNA vaccines, one was not prescribed any treatment but fully recovered by week 3 [39]; one presented winging of the scapula but denied any pain, dysfunction, or disruption from activity at three months only with physical therapy [27]. In one case, the pain resolved, but the patient continued to present hand weakness at a three-month follow-up [50]. Resolution of symptoms within two months after rehabilitation was reported in one patient [47]. However, in another case, receiving physical therapy and electric stimulation, muscle weakness persisted over four months [50]. In patients with PTS after a viral vector vaccine, two cases did not receive any medication; they were recommended only physical therapy, but the outcome was not reported [23,31]. One PTS case had a full recovery within two months, while one patient had a poor recovery by week 4 without treatment [39]. 

Corticosteroids were administered in 65.7% of patients receiving mRNA vaccines and 66.7% of cases with viral vector vaccines. Notably, a review on PTS after SARS-CoV-2 infection found that only 46.2% of cases received steroids [62]. The authors used different doses and regimens, with outcomes from complete recovery to minimal improvement of symptoms. Nevertheless, it is essential to conduct randomized, placebo-controlled studies in order to assess the impact of steroids and other interventions on individuals with PTS. Rehabilitation was recommended for 20% and 43.8% of cases, respectively, similar to patients with PTS following SARS-CoV-2 infection. 

Among patients with mRNA vaccination, full recovery was noted for 12.1% of cases, while 25% of individuals with previous viral vector vaccination completely recovered. In patients with previous SARS-CoV-2 infection, 26.3% had a complete remission by six months [62]. Nonetheless, the follow-up duration was variable, and firm conclusions on the prognosis cannot be drawn. 

The causality assessment found that 32 cases were possibly caused by vaccination. None of the included cases met the WHO-UMC scale criteria on withdrawal (“Response to withdrawal clinically reasonable”) to be marked as “Probable/likely”. However, this item does not apply to vaccines. Prior studies have demonstrated that the notion of “rechallenge”, typically employed in the evaluation of causality in pharmaceuticals, has proven helpful for specific vaccine incidents, such as GBS following tetanus toxoid vaccination, where GBS occurred on three distinct occasions in a single patient within a few weeks of tetanus toxoid administration [20]. In patients receiving mRNA vaccines, two cases presented with PTS symptoms, aggravated by the second dose [37,45]. In another case, PTS recurred after a subsequent influenza vaccine, administered six months later; the exact symptoms returned, but with reduced intensity, and disappeared spontaneously after a week [60]. Within the group of viral vector vaccines, one patient received the second administration of the COVID-19 immunization without experiencing any further adverse reactions [23]. Another case presented with dyspnea after the first dose, which improved over the following weeks. However, within a week after the second dose, dyspnea reappeared with increased severity [32]. Therefore, it is essential to be aware of the possibility of PTS recurrence with subsequent similar or unrelated vaccines. Furthermore, the cases with unequivocal reappearance or worsening of the PTS symptoms after rechallenge further support the hypothesis that both vaccine types can have this particular adverse event [81]. 

Causality assessment of AEFIs may be performed at various levels. First, at the level of the individual AEFI case report, the assessment estimates the probability that the development of an AEFI in a particular individual is causally linked to the use of vaccines. Establishing a certain causal association between an individual AEFI and a specific vaccination based on a single AEFI case report is often unattainable. Nevertheless, it is crucial not to overlook the case reports of AEFI as they might act as signals and prompt hypotheses on a connection between vaccination and the particular event in question. These hypotheses may then be tested in specific studies to determine whether there is a causal relationship. The pooling of data on individual patients is valuable for formulating assumptions. The instance of the rota-virus vaccination and intussusception serves as a noteworthy example [20]. Furthermore, while examining signals, evaluating whether a specific vaccine is likely to result in a particular adverse event takes into account all available information from individual AEFI cases, structured data-collecting systems, and, where relevant, cluster studies and non-clinical data [82].

Nonetheless, it is essential to try evaluating this association in order to identify a possible new vaccine-related reaction. Our aim of causality assessment at the individual level was to address the question, “Did the COVID-19 vaccine given to a particular individual cause PTS?”. Obtaining a definitive answer to this question is rarely feasible. Therefore, in the majority of cases, the evaluation entails a systematic investigation of all potential factors contributing to an AEFI in order to determine whether the evidence supports the vaccine as a cause, contradicts this conclusion, or remains uncertain [20].

Clinical or laboratory proof, most often found for live attenuated vaccines, is definitive proof that the vaccine caused the event. For example, in the case of aseptic meningitis after vaccination with the Urabe mumps vaccine virus, the detection of the Urabe virus in CSF provided conclusive evidence that it was the causative agent of the meningitis. 

According to the WHO [20], in the case of a consistent temporal relationship but insufficient evidence for causality (it may be a new vaccine-linked event), the information on new vaccine-related events should be recorded in a national database. As time progresses, the administration of more similar vaccines and the collection of similar events from one or multiple sources will contribute to the identification of a signal indicating a potential new causal relationship, or a novel aspect of an existing association, between a vaccine and an event or a group of interconnected events. Also, the causal relationship may be modified as new information emerges on the same or similar events [20]. For example, a report of narcolepsy following the administration of the AS03-adjuvanted H1N1 influenza Pandemrix^®^ vaccine can currently be categorized as a likely adverse event related to vaccine products. Prior to the establishment of the association between narcolepsy and the influenza vaccine in 2010 through scientific evidence, the same case would have been considered coincidental or indeterminate [83].

Therefore, the cases in our review provide data on the possibility that COVID-19 vaccines might cause PTS. The collection of reports pertaining to such events has significance as they may eventually be regarded as a signal and give rise to hypotheses about a potential link between a vaccination and the incident in question. Consequently, specific studies could be designed to examine the existence of a causal relationship [82].

The limitations of the current review are mainly related to the quality of the included studies, with missing, insufficient, or ambiguous descriptions of the data. This could be due to substantial methodological variation in SARS-CoV-2 studies and the need for standardized methodology and precise reporting criteria. Furthermore, other PTS triggers, like infections, intravenous maneuvers (i.e., intravenous therapy, contrast administration, blood withdrawal), and certain medications, are not thoroughly assessed in the included studies. Although case reports present an increased possibility of bias, they are essential in advancing knowledge, particularly for rare conditions. 

We compared the clinical and ancillary investigation results for patients receiving mRNA and viral vector COVID-19 vaccines. However, our results could be biased by a lack of data on the characteristics of the populations in which they were administered. Future studies should control for demographic and other confounding factors to gain more reliable insights into the differences in adverse events between mRNA and viral vector vaccines. This will be essential for informing vaccination decisions and optimizing vaccine strategies.

Also, we used Fisher’s exact test to determine if there was a significant difference between the two vaccine subgroups. Fisher’s exact test is mainly employed when sample sizes are limited, which inevitably raises another issue about its application. Regardless of the outcome of a statistical test, one cannot have a substantial level of confidence in findings derived from small sample sizes. Tests conducted on such data will exhibit low statistical power to reject the null hypothesis, and the chance of such a sample being representative of a population is low. Further research, including larger sample sizes, is required to evaluate the differences between groups of patients with PTS after receiving mRNA and viral vector vaccines. In addition, although it would be interesting to compare the current results with PTS resulting from specific vaccinations such as influenza, pertussis, typhoid, diphtheria, tetanus, smallpox, or human papillomavirus, it is worth noting that no cohort studies have been conducted on a single vaccine to yet. 

Another limitation of the present work is that we could not analyze the epidemiology of PTS following SARS-CoV-2 vaccination. However, previous research aimed to investigate the link between neuralgic amyotrophy and COVID-19 vaccination using the World Health Organization’s global pharmacovigilance database (VigiBase) [84]. Of 1,731,147 adverse drug reactions (ADR) reports related to COVID-19 vaccines, the authors identified 335 (0.02%) neuralgic amyotrophy cases. The research identified a correlation between PTS and mRNA-based COVID-19 vaccinations as well as the ChAdOx1 nCoV-19 vaccine. However, the level of disproportionality observed was not greater than that seen with influenza vaccines. The association between PTS and mRNA-based COVID-19 vaccinations was stronger compared to the ChAdOx1 nCoV-19 vaccine [84]. Nonetheless, ADRs in countries where reports are not linked to VigiBase might have been omitted. Besides missing data, further limitations of the use of VigiBase include various biases. The database contains heterogeneous information, and the system depends on national centers for the timeliness, completeness, and quality of reports [85]. Nonetheless, by monitoring and examining real-life data collected from the French Network of Regional PharmacoVigilance Centers (RFCRPV), researchers were able to detect pharmacovigilance signals, such as PTS. From the start of the COVID-19 immunization campaign in France until 10 February 2022, a total of 59 PTS cases were documented. Among these cases, 43 were attributed to tozinameran, while 16 were associated with elasomeran [86]. An experienced pharmacologist and neurologist were commissioned to evaluate the neurological events resulting from COVID-19 vaccination surveillance. The cases were thoroughly studied and assessed. The diagnosis was confirmed in a total of 30 individuals. In 29 instances, the diagnosis was not definitively established due to either an incompatible delay in the onset or incomplete evidence. Eight cases presented particular forms, including a relapse, contralateral PTS, post-partum PTS, and three cases arising within the setting of trauma or strenuous work. The findings indicate that the vaccine’s role cannot be disregarded [86].

Despite the methodological constraints, observing individual patients provides important insights into etiology, pathogenesis, natural evolution, and possible treatments [18]. Case reports and case studies describe new events, being the first-line evidence to further hypothesis testing with statistical approaches. 

Current evidence suggests that PTS may occur after all COVID-19 vaccine types, with some differences between subgroups. Also, a prerequisite of a high index of suspicion of PTS in patients with previous COVID-19 vaccination is necessary, as clinical manifestations can be variable. Furthermore, a standardized approach is needed when investigating and reporting on PTS, with a comprehensive assessment of patients. 

## Figures and Tables

**Table 1:** Demographics of patients with Parsonage–Turner syndrome.

Age at Diagnosis (Years)	mRNA Vaccine (%)	Viral Vector Vaccine (%)	*p*-Value *
0–10	0 (0%)	0 (0%)	N/A
11–20	2 (5.6%)	0 (0%)	0.5472
21–30	2 (5.6%)	2 (11.1%)	0.5936
31–40	5 (13.9%)	4 (22.2%)	1.0000
41–50	15 (41.7%)	1 (5.6%)	**0.0098**
51–60	5 (13.9%)	2 (11.1%)	1.0000
61–70	2 (5.6%)	7 (38.9%)	**0.0041**
>71	5 (13.9%)	2 (11.1%)	1.0000
**Gender**			
Male	22 (61.1%)	15 (83.3%)	0.1272
Female	14 (38.9%)	3 (16.7%)	0.1272

* The results with statistical significance (*p* < 0.05) are marked in bold.

**Table 2:** Comorbidities of patients with Parsonage–Turner syndrome.

Medical History	mRNA Vaccine n (%)	Viral Vector Vaccine n (%)	*p*-Value
Hypertension	2/17 (11.8%)	2/9 (22.2%)	0.5906
Hyperlipidemia	1/17 (5.9%)	2/9 (22.2%)	0.2677
Coronary artery disease	1/17 (5.9%)	0/9 (0%)	1.0000
Diabetes	0/17 (0%)	1/9 (11.1%)	0.3462
Smoking	1/17 (5.9%)	1/9 (11.1%)	1.0000
Malignancies	1/17 * (5.9%)	0/9 (0%)	1.0000
Previous COVID-19	1/17 (5.9%)	0/9 (0%)	1.0000
Previous Lyme disease	1/17 (5.9%)	0/9 (0%)	1.0000
Celiac disease	0/17 (0%)	1/9 (11.1%)	0.3462
None	9/17 (52.9%)	5/9 (55.6%)	1.0000
Not reported	19/36 (52.8%)	9/18 (50.0%)	N/A

* Humerus osteosarcoma s/p resection with hemiarthroplasty at age 15 on the same side with PTS.

**Table 3:** Clinical assessment of patients with PTS.

	mRNA Vaccine n (%)	Viral Vector Vaccine n (%)	*p*-Value *
**Duration since vaccination, days**			
0–14	27/36 (75%)	15/17 (88.2%)	0.4694
15–30	8/36 (22.2%)	2/17 (11.8%)	0.4711
31–90	1/36 (2.8%)	0/17 (0%)	1.0000
Indeterminable	0/36 (0%)	1/18 (5.6%)	N/A
**PTS onset**			
Sudden	25/29 (86.2%)	8/10 (80%)	0.6360
Progressive	2/29 (6.9%)	2/10 (20%)	0.2670
Indeterminable	7/36 (19.4%)	8/18 (44.4%)	N/A
**Localization**			
Unilateral	34/36 (94.4%)	17/17 (100%)	1.0000
Bilateral	2/36 (5.6%)	0/17 (0%)	1.0000
Indeterminable	0/36 (0%)	1/18 (5.6%)	N/A
Ipsilateral to vaccination site	21/28 (75%)	11/12 (91.7%)	0.3955
Contralateral to vaccination site	7/28 (25%)	1/12 (8.3%)	0.3955
Indeterminable/vaccination site not reported	8/36 (22.2%)	6/18 (33.3%)	N/A
**Pain**	34/35 (97.1%)	12/13 (92.3%)	0.4725
**Motor deficit**	35/36 (97.2%)	16/17 (94.1%)	0.5428
**Muscle wasting**	9/30 (30%)	9/11 (81.8%)	**0.0046**
**Paresthesia**	15/30 (50%)	3/11 (27.3%)	0.2911
**Sensory loss**	10/30 (33.3%)	5/13 (38.5%)	0.7422
**Other neurological involvement**			
Lower limbs	0/36 (0%)	1/18 (5.6%)	0.3333
Diaphragm	0/36 (0%)	2/18 (11.1%)	0.1069

* The results with statistical significance (*p* < 0.05) are marked in bold.

**Table 4:** Ancillary testing.

	mRNA Vaccine n (%)	Viral Vector Vaccine n (%)	*p*-Value
**CSF**			
Albuminocytological dissociation	1/3 (33.3%)	3/3 * (100%)	0.4000
Normal	2/3 (66.7%)	0/3 (0%)	0.4000
Not reported/not conducted	33/36 (91.7%)	15/18 (83.3%)	N/a
**MRI of cervical/lumbar spine**			
Normal	16/20 (80%)	4/6 (66.7%)	0.5960
Degenerative changes	4/20 (20%)	2/6 ** (33.3%)	0.5960
**MRI of brachial/lumbar plexus**			
Normal	7/18 (38.9%)	3/9 (33.3%)	1.0000
Muscle edema	5/18 (27.8%)	4/9 (44.4%)	0.4228
Edematous nerves	4/18 (22.2%)	0/9 (0%)	0.4228
Local lymphadenopathy	3/18 (16.7%)	4/9 (44.4%)	0.1751
Increased nerve signal	4/18 (22.2%)	3 **/9 (33.3%)	0.6527
Hourglass constrictions	2/18 (11.1%)	0/9 (0%)	0.5385
**Nerve conduction study**			
Normal	5/23 (21.7%)	1/10 (10%)	0.6402
Pathological findings	18/23 (78.3%)	9/10 (90%)	0.6402
**Electromyography**			
Normal	3/26 (11.5%)	0/11 (0%)	0.5399
Pathological	23/26 (88.5%)	11/11 (100%)	0.5399
**Unspecified electrodiagnostic study**			
Pathological findings	7/7 (100%)	7/7 (100%)	1.0000

Notes: CSF: cerebrospinal fluid. MRI: magnetic resonance imaging. * One patient also presented with Guillain Barre syndrome. ** One patient presented lumbar PTS.

**Table 5:** Interventions and outcomes of Parsonage–Turner syndrome.

	mRNA Vaccine n (%)	Viral Vector Vaccine n (%)	
**Treatment**			
Reported	35/36 (97.2%)	16/18 (88.9%)	
Not reported	1/36 (2.8%)	2/18 (11.1%)	
Received medical treatment	32/35 (91.4%)	12/16 (75%)	
No medication	5/35 (14.3%)	4/16 (25%)	
Physical/occupational therapy	14/35 (20%)	7/16 (43.8%)	
Corticosteroids	23/35 (65.7%)	8/12 (66.7%)	
Intravenous immunoglobulin	1/35 (2.9%)	0/12 (0%)	
Nonsteroidal anti-inflammatory drugs	7/35 (20%)	4/12 (33.3%)	
Pregabalin	5/35 (14.3%)	2/12 (16.7%)	
Gabapentin	5/35 (14.3%)	2/12 (16.7%)	
Amitriptyline/Nortriptyline	2/35 (5.7%)	0/12 (0%)	
Opiates	4/35 (11.4%)	0/12 (0%)	
Unspecified analgetic treatment	2/35 (5.7%)	1/12 (8.3%)	
Acetaminophen	1/35 (2.9%)	1/12 (8.3%)	
Continuous positive airway pressure	0/35 (0%)	1/12 * (8.3%)	
Surgery	1/35 (2.9%)	0/12 (0%)	
**Follow-up duration**			
1–30 days	3/28 (10.7%)	3/13 (23.1%)	
31–60 days	10/28 (35.7%)	2/13 (15.4%)	
>60 days	15/28 (53.6%)	8/13 (61.5%)	
Unclear	8/36 (22.2%)	5/18 (27.8%)	
**Evolution**			***p*-value**
Full recovery	4/33 (12.1%)	3/12 (25%)	0.3619
Improvement	28/33 (84.8%)	9/12 * (75%)	0.6609
Worsening	1/33 (3.0%)	0/12 (0%)	1.0000
Unclear	3/36 (8.3%)	6/18 (33.3%)	N/A

Notes: * Including patients with diaphragmatic dysfunction.

## Data Availability

All relevant data are available within the article.

## Supplementary Materials

The following supporting information can be downloaded at https://www.mdpi.com/article/10.3390/vaccines12030306/s1. Table S1: Characteristics of the included studies. Table S2: Clinical characteristics of PTS patients. Table S3. Causality assessment.
