# Supplementary material for: Parsonage–Turner Syndrome following COVID-19 Vaccination: A Systematic Review
Source: Vaccines (Basel). 2024 Mar 14;12(3):306. doi: 10.3390/vaccines12030306 (PMC10975425; doi:10.3390/vaccines12030306)
Supplement: Supplementary file 1 [file vaccines-12-00306-s001.zip › Table S2. Clinical characteristics of PTS patients.pdf]

S2. Clinical characteristics of Parsonage Turner syndrome patients

Study	Pain	Motor deficit	Muscle wasting	Paresthesia	Sensory loss	Notes
Amjad 2022	Yes	Yes	No	No	No	Bilateral
Balloy 2021	Yes	Yes	Yes	No	No	Ipsilateral
Bernheimer 2022	Yes	Yes	Yes	Yes	No	Unilateral
Cabona 2022	Yes	Yes	Yes	No	Yes	Ipsilateral, associated with torsional neuropathy
Cascio 2022	Yes	Yes	No	No	No	Contralateral
Cassart 2023	Yes	Yes	Yes	No	Yes	Unilateral
Chen 2022	Yes	Yes	Yes	No	No	Contralateral
Chua 2022	Yes	Yes	No	Yes	Yes	Ipsilateral
Civardi 2022	Yes	Yes	No	No	Yes	Unilateral
Coffman 2021	Yes	Yes	Yes	Yes	No	Ipsilateral
Crespo Burillo 2021	Yes	No	No	No	No	Unilateral, including phrenic nerve
Diazsegarra 2022	No	Yes	Yes	Yes	Yes	Contralateral, without pain
Dovgan 2022	Yes	Yes	Yes	Yes	Yes	Unilateral, associated with GBS
Igbokwe 2022	Yes	Yes	No	No	No	Unilateral
Flikkema 2021	Yes	Yes	No	Yes	Yes	Ipsilateral
Fukahori 2023	Yes	Yes	No	No	Yes	Ipsilateral
Ishizuka 2023	Yes	Yes	Yes	No	No	Unilateral
Joe James 2022	Yes	Yes	Yes	No	Yes	Ipsilateral
Kang 2022	Yes	Yes	No	No	No	Phrenic nerve
Kim 2021	No	Yes	Yes	Yes	Yes	Unilateral, lumbosacral plexus
Koduri 2022	Yes	Yes	No	No	No	Unilateral
Koh 2021, case 1	Yes	Yes	No	Yes	No	Ipsilateral
Koh 2021, case 2	Yes	Yes	No	Yes	No	Contralateral
Koh 2021, case 3	Yes	Yes	No	Yes	No	Ipsilateral
Lakkireddy 2022	Yes	Yes	Yes	Yes	No	Contralateral
Leemans 2022	Yes	Yes	No	No	Yes	Unilateral
Loganathan 2023	Yes	Yes	No	Yes	No	Ipsilateral

Mahajan 2021	Yes	Yes	No	No	No	Ipsilateral, aggravated by the 2nd dose
Meixedo 2023	Yes	Yes	Yes	No	No	Ipsilateral
Mejri 2022	Yes	Yes	Yes	Yes	No	Ipsilateral
Min 2022, case 1	N/R	Yes	N/R	N/R	N/R	Ipsilateral
Min 2022, case 2	Yes	Yes	N/R	N/R	N/R	Ipsilateral
Min 2022, case 3	Yes	Yes	N/R	N/R	N/R	Ipsilateral
Min 2022, case 4	N/R	Yes	N/R	N/R	N/R	Ipsilateral
Min 2022, case 5	N/R	Yes	N/R	N/R	N/R	Ipsilateral
Min 2022, case 6	N/R	Yes	N/R	N/R	N/R	Ipsilateral
Min 2022, case 7	N/R	Yes	N/R	N/R	N/R	Ipsilateral
Min 2022, case 8	Yes	Yes	N/R	N/R	N/R	Contralateral
Min 2022, case 9	Yes	Yes	N/R	N/R	N/R	Ipsilateral
Min 2022, case 10	Yes	No	N/R	N/R	N/R	Ipsilateral
Min 2022, case 11	Yes	Yes	N/R	N/R	N/R	Bilateral
Min 2022, case 12	Yes	Yes	N/R	N/R	N/R	Ipsilateral
Oncel 2022	Yes	Yes	No	No	No	Ipsilateral
Pham 2022	Yes	Yes	No	Yes	No	Unilateral
Pilgram 2021	Yes	Yes	Yes	No	No	Unilateral
Queler 2022, case 1	Yes	Yes	Yes	Yes	No	Contralateral
Queler 2022, case 2	Yes	Yes	No	Yes	Yes	Ipsilateral
Sharma R 2022	Yes	Yes	Yes	No	Yes	Ipsilateral
Sharma A 2022	Yes	Yes	Yes	No	No	Unilateral
Shields 2022, case 1	Yes	Yes	No	No	No	Ipsilateral
Shields 2022, case 2	Yes	Yes	No	No	No	Ipsilateral
Shields 2022, case 3	Yes	Yes	No	No	Yes	Ipsilateral
Shields 2022, case 4	Yes	Yes	No	Yes	No	Ipsilateral
Shields 2022, case 5	Yes	Yes	No	No	No	Contralateral
Shields 2022, case 6	Yes	Yes	No	No	No	Ipsilateral

Van Boxstael 2022	N/R	N/R	N/R	N/R	N/R	N/R
Van Lancker 2022	Yes	Yes	Yes	Yes	No	Contralateral
Vitturi 2021	Yes	Yes	Yes	No	Yes	Ipsilateral
Yeoh 2023	Yes	Yes	Yes	Yes	Yes	Ipsilateral
