# Supplementary material for: Parsonage–Turner Syndrome following COVID-19 Vaccination: A Systematic Review
Source: Vaccines (Basel). 2024 Mar 14;12(3):306. doi: 10.3390/vaccines12030306 (PMC10975425; doi:10.3390/vaccines12030306)
Supplement: Supplementary file 1 [file vaccines-12-00306-s001.zip › Table S3. Causality assessment.pdf]

Table S3. Case causality assessment.

Study	Certain	Probable / Likely	Possible	Unlikely	Conditional / Unclassified	Unassessable/ Unclassifiable	Notes
Amjad 2022			Yes*				Rechallenge not done. The PTS occurred after the 2nd dose. No information is provided regarding any AEFI after the 1st dose. Could also be explained by disease or other drugs, although unlikely. “Response to withdrawal clinically reasonable” criteria not met.
Balloy 2021						Yes	Differential diagnosis not reported. Could also be explained by disease or other drugs. Rechallenge: the patient received the second injection of the COVID-19 vaccination (AZD1222) with no additional side effect. More data for proper assessment needed.
Bernheimer 2022			Yes				Differential diagnosis not reported. Could also be explained by disease or other drugs. The PTS occurred after the 2nd dose. No information is provided regarding any AEFI after the 1st dose.
Cabona 2022			Yes				Differential diagnosis not reported. Could also be explained by disease or other drugs. No data on vaccine type/dose.
Cascio 2022						Yes	Differential diagnosis not reported. Could also be explained by disease or other drugs. More data for proper assessment needed (no neurophysiological investigations, no brachial plexus MRI). No rechallenge.
Cassart 2023			Yes				Incomplete differential diagnosis (e.g., HIV test not reported). Could also be explained by disease or other drugs. Rechallenge: hypoesthesia in the left shoulder after 1st dose. Weakness and pain at 3 weeks after 2nd dose. Rechallenge (2nd dose): aggravation of symptoms.
Chen 2022			Yes				Differential diagnosis not reported. Could also be explained by disease or other drugs. No information is provided regarding any AEFI after the 1st dose.
Chua 2022			Yes				Differential diagnosis not reported (Could also be explained by disease or other drugs). PTS developed after 2nd dose.
Civardi 2022			Yes*				Rechallenge not done. PTS developed after 1st dose. “Response to withdrawal clinically reasonable” criteria not met.
Coffman 2021			Yes				Differential diagnosis not reported (Could also be explained by disease or other drugs). The PTS occurred after the 2nd dose. No information is provided regarding any AEFI after the 1st dose.

Crespo Burillo 2021			Yes				Differential diagnosis not reported (Could also be explained by disease or other drugs). No data on vaccine dose.
Diazsegarra 2022			Yes				Incomplete differential diagnosis (e.g., HIV test not reported). Could also be explained by disease or other drugs. No data on vaccine dose.
Dovgan 2022						Yes	Incomplete differential diagnosis (e.g., HIV test not reported). Could also be explained by disease or other drugs. Concomitant Guillain Barre syndrome. More data for proper assessment needed. No data on vaccine dose.
Flikkema 2021			Yes				Incomplete differential diagnosis (e.g., blood tests not reported). Could also be explained by disease or other drugs. Rechallenge not done. PTS developed after 1st dose.
Fukahori 2023			Yes			?	Incomplete differential diagnosis (e.g., HIV test not reported). Could also be explained by disease or other drugs. Anti-GM1 IgG, and IgM antibodies detected in the serum. PTS developed after 2nd dose.
Igbokwe 2022			Yes				Incomplete differential diagnosis (e.g., HIV test not reported). Could also be explained by disease or other drugs. No data on vaccine type/dose.
Ishizuka 2023			Yes				Differential diagnosis not reported (Could also be explained by disease or other drugs). The PTS occurred after the 2nd dose. No information is provided regarding any AEFI after the 1st dose.
Joe James 2022			Yes				Incomplete differential diagnosis (e.g., Hepatitis E, neuroborreliosis). Could also be explained by disease or other drugs. Rechallenge not done. PTS developed after 1st dose.
Kang 2022			Yes*				Rechallenge positive. Incomplete differential diagnosis (e.g., infection, vasculitis). Could also be explained by disease or other drugs, although unlikely.“Response to withdrawal clinically reasonable” criteria not met.
Kim 2021			Yes*				Rechallenge not done. No data on vaccine dose. Could also be explained by disease or other drugs, although unlikely. “Response to withdrawal clinically reasonable” criteria not met.
Koduri 2022			Yes				Incomplete differential diagnosis (e.g., blood tests not reported). Could also be explained by disease or other drugs. PTS developed after 2nd dose.
Koh 2021, case 1			Yes				Unclear what other triggers of PTS were investigated. Could also be explained by disease or other drugs. PTS developed after 1st dose.
Koh 2021, case 2			Yes				Unclear what other triggers of PTS were investigated. Could also be explained by disease or other drugs. The PTS occurred after the 2nd

							dose. No information is provided regarding any AEFI after the 1st dose.
Koh 2021, case 3			Yes				Unclear what other triggers of PTS were investigated. Could also be explained by other disease or other drugs. The PTS occurred after the 2nd dose. No information is provided regarding any AEFI after the 1st dose.
Lakkireddy 2022			Yes				Incomplete differential diagnosis (e.g., Hepatitis E, neuroborreliosis). Could also be explained by other disease or other drugs. Insidious symptoms. No data on vaccine dose.
Leemans 2022						Yes	Differential diagnosis not reported (Could also be explained by disease or other drugs). Lower trunk brachial plexopathy. More data for proper assessment needed. The PTS occurred after the 2nd dose. No information is provided regarding any AEFI after the 1st dose.
Loganathan 2023			Yes				Incomplete differential diagnosis (e.g., HIV, neuroborreliosis). Could also be explained by other disease. PTS occurred after the 4th dose. The first 3 doses were Pfizer vaccines. No information is provided regarding any AEFI after the previous doses.
Mahajan 2021			Yes*				Rechallenge positive (pain after first dose, that flared after the second dose). Incomplete differential diagnosis (e.g., HIV). Could also be explained by disease or other drugs, although unlikely. "Response to withdrawal clinically reasonable" criteria not met.
Meixedo 2023						Yes	Ultrasound (shoulder, 2 months): partial insertional tear of the supraspinatus and exuberant left subacromial bursitis. MRI: tendon pathology. Differential diagnosis not reported. Could also be explained by disease or other drugs. Limited clinical information. More data for proper assessment needed. No data on vaccine dose.
Mejri 2022			Yes				Unclear what other triggers of PTS were investigated. Could also be explained by disease or other drugs. PTS occurred after the 2nd dose. No pain after the 1st dose.
Min 2022, case 1						Yes	Differential diagnosis not reported (Could also be explained by disease or other drugs). Limited clinical information. More data for proper assessment needed. No data on vaccine dose.
Min 2022, case 2						Yes	Differential diagnosis not reported (Could also be explained by disease or other drugs). Limited clinical / paraclinical information. More data for proper assessment needed. No data on vaccine dose.
Min 2022, case 3						Yes	Differential diagnosis not reported (Could also be explained by disease or other drugs). Limited clinical / paraclinical information.

							More data for proper assessment needed. PTS developed after 1st dose.
Min 2022, case 4						Yes	Differential diagnosis not reported (Could also be explained by disease or other drugs). Limited clinical / paraclinical information. More data for proper assessment needed. PTS developed after 1st dose.
Min 2022, case 5						Yes	Differential diagnosis not reported (Could also be explained by disease or other drugs). Limited clinical / paraclinical information. More data for proper assessment needed. PTS developed after 1st dose.
Min 2022, case 6						Yes	Differential diagnosis not reported (Could also be explained by disease or other drugs). Limited clinical / paraclinical information. More data for proper assessment needed. The PTS occurred after the 2nd dose. No information is provided regarding any AEFI after the 1st dose.
Min 2022, case 7						Yes	Differential diagnosis not reported (Could also be explained by disease or other drugs). Limited clinical / paraclinical information. More data for proper assessment needed. Cross-vaccination (AstraZeneca and then Pfizer)
Min 2022, case 8						Yes	Differential diagnosis not reported (Could also be explained by disease or other drugs). Limited clinical / paraclinical information. More data for proper assessment needed. PTS developed after 1st dose.
Min 2022, case 9						Yes	Differential diagnosis not reported (Could also be explained by disease or other drugs). Limited clinical / paraclinical information. More data for proper assessment needed. PTS developed after 1st dose.
Min 2022, case 10						Yes	Differential diagnosis not reported (Could also be explained by disease or other drugs). Limited clinical / paraclinical information. More data for proper assessment needed. PTS developed after 1st dose.
Min 2022, case 11						Yes	Differential diagnosis not reported (Could also be explained by disease or other drugs). Limited clinical / paraclinical information. More data for proper assessment needed. PTS developed after 1st dose.
Min 2022, case 12						Yes	Differential diagnosis not reported (Could also be explained by disease or other drugs). Limited clinical / paraclinical information.

							More data for proper assessment needed. PTS developed after 1st dose.
Oncel 2022			Yes				Incomplete differential diagnosis (e.g., Hepatitis E, neuroborreliosis). Could also be explained by disease or other drugs. The PTS occurred after the 2nd dose. No information is provided regarding any AEFI after the 1st dose.
Pham 2022						Yes	Incomplete differential diagnosis (e.g., Hepatitis E, neuroborreliosis). Could also be explained by disease or other drugs. Three weeks prior to symptom onset, the patient experienced COVID-like symptoms but had a negative rapid antigen test. A diagnosis of cervical radiculopathy and PTS was made, with viral infection followed by vaccination as the suspected etiology. More data for proper assessment needed. The PTS occurred after the 3rd dose. No information is provided regarding any AEFI after the 1st and 2nd dose.
Pilgram 2021						Yes	Differential diagnosis not reported. Could also be explained by disease or other drugs. Electrodiagnostic study (2 months after onset): mononeuritis multiplex. More data for proper assessment needed. No data on vaccine type. PTS developed after 1st dose.
Queler 2022, case 1			Yes				Vaccination alone or a combination of vaccination and recent infection (Lyme disease) may have been a trigger. PTS developed after 1st dose.
Queler 2022, case 2			Yes				Incomplete differential diagnosis (e.g., blood tests not reported). Could also be explained by disease or other drugs. PTS developed after 1st dose.
Sharma R 2022						Yes	Rechallenge: uneventful. Incomplete differential diagnosis (e.g., HIV, Hepatitis E, neuroborreliosis). Could also be explained by disease or other drugs. More data for proper assessment needed. PTS developed after 1st dose.
Sharma A 2022			Yes				Incomplete differential diagnosis (e.g., infections). Could also be explained by disease or other drugs. No data on vaccine dose.
Shields 2022, case 1						Yes	Unclear what other triggers of PTS were investigated. Could also be explained by disease or other drugs. Limited clinical / paraclinical information. More data for proper assessment needed. PTS developed after 1st dose
Shields 2022, case 2						Yes	Unclear what other triggers of PTS were investigated. Could also be explained by disease or other drugs. Limited clinical / paraclinical information. More data for proper assessment needed. The PTS

							occurred after the 2nd dose. No information is provided regarding any AEFI after the 1st dose.
Shields 2022, case 3						Yes	Unclear what other triggers of PTS were investigated. Could also be explained by disease or other drugs. Limited clinical / paraclinical information. More data for proper assessment needed. The PTS occurred after the 2nd dose. No information is provided regarding any AEFI after the 1st dose.
Shields 2022, case 4						Yes	Unclear what other triggers of PTS were investigated. Could also be explained by disease or other drugs. Limited clinical / paraclinical information. More data for proper assessment needed. PTS developed after 1st dose.
Shields 2022, case 5						Yes	Unclear what other triggers of PTS were investigated. Could also be explained by disease or other drugs. The PTS occurred after the 2nd dose. No information is provided regarding any AEFI after the 1st dose.
Shields 2022, case 6						Yes	Unclear what other triggers of PTS were investigated. Could also be explained by disease or other drugs. Limited clinical / paraclinical information. More data for proper assessment needed. The PTS occurred after the 2nd dose. No information is provided regarding any AEFI after the 1st dose.
Van Boxstael 2022						Yes	Unclear what other triggers of PTS were investigated. (Could also be explained by disease or other drugs). Limited clinical / paraclinical information. More data for proper assessment needed. The PTS occurred after the 2nd dose. No information is provided regarding any AEFI after the 1st dose.
Van Lancker 2022			Yes				PTS recurred after subsequent influenza vaccination. Differential diagnosis not reported. Could also be explained by disease or other drugs. The PTS occurred after the 2nd dose. The patient had isolated persistent mild pain at the injection<br>Site after the 1st dose.
Vitturi 2021			Yes				Incomplete differential diagnosis (e.g., blood tests not reported). Could also be explained by other disease. PTS developed after 1st dose.
Yeoh 2023			Yes				Incomplete differential diagnosis (e.g., HIV, Hepatitis E, neuroborreliosis). Could also be explained by disease or other drugs. Cross-vaccination; 2 doses of an inactivated COVID-19 vaccine 7 months before mRNA booster. The PTS occurred after the 3rd vaccination. No information is provided regarding any AEFI after the 1st and 2nd dose.

* The case has a thorough assessment of possible PTS triggers. However, it does not meet the WHO-UMC scale criteria on withdrawal ("Response to withdrawal clinically reasonable") to be marked as "Probable/likely."
